# Genome-Wide Association Analyses Reveal Genomic Regions Controlling Canopy Wilting in Soybean

**DOI:** 10.1534/g3.119.401016

**Published:** 2020-02-28

**Authors:** Clinton J. Steketee, William T. Schapaugh, Thomas E. Carter, Zenglu Li

**Affiliations:** * Institute of Plant Breeding, Genetics, and Genomics and Department of Crop and Soil Sciences, The University of Georgia, Athens, GA, 30602; † Department of Agronomy, Kansas State University, Manhattan, KS, 66506; ‡ Department of Crop and Soil Sciences, North Carolina State University, USDA-ARS, Raleigh, NC, 27607

**Keywords:** soybean, *Glycine max*, drought tolerance, canopy wilting, genome-wide association study (GWAS)

## Abstract

Drought stress causes the greatest soybean [*Glycine max* (L.) Merr.] yield losses among the abiotic stresses in rain-fed U.S. growing areas. Because less than 10% of U.S. soybean hectares are irrigated, combating this stress requires soybean plants which possess physiological mechanisms to tolerate drought for a period of time. Phenotyping for these mechanisms is challenging, and the genetic architecture for these traits is poorly understood. A morphological trait, slow or delayed canopy wilting, has been observed in a few exotic plant introductions (PIs), and may lead to yield improvement in drought stressed fields. In this study, we visually scored wilting during stress for a panel of 162 genetically diverse maturity group VI-VIII soybean lines genotyped with the SoySNP50K iSelect BeadChip. Field evaluation of canopy wilting was conducted under rain-fed conditions at two locations (Athens, GA and Salina, KS) in 2015 and 2016. Substantial variation in canopy wilting was observed among the genotypes. Using a genome-wide association mapping approach, 45 unique SNPs that tagged 44 loci were associated with canopy wilting in at least one environment with one region identified in a single environment and data from across all environments. Several new soybean accessions were identified with canopy wilting superior to those of check genotypes. The germplasm and genomic regions identified can be used to better understand the slow canopy wilting trait and be incorporated into elite germplasm to improve drought tolerance in soybean.

Soybean [*Glycine max* (L.) Merrill] is the world’s leading oilseed crop used to produce vegetable oil, protein feed for livestock, biodiesel, and many soyfoods. Drought stress is the most significant abiotic threat to the agricultural productivity of soybean around the world, and can reduce yield by more than 40% (Specht *et al.* 1999; Purcell and Specht 2004).

Loss of turgor and leaf droop, known commonly as canopy wilting, is an often-observed response to drought stress in soybean. Some exotic soybean types exhibit a slow or delayed canopy wilting response to drought, which may reflect favorable underlying plant mechanisms to access soil moisture, conserve soil moisture prior to stress, or use water more efficiently. One mechanism related to water conservation is to restrict transpiration early in the growing season whenever vapor pressure deficit (VPD) is high, so that plants can utilize saved soil water during pod filling when drought stress in soybean is usually more detrimental to yield. Plant introduction (PI) 416937 is a Japanese maturity group (MG) VI introduction identified in the 1980s as exhibiting slower wilting under water deficit conditions than existing cultivars (Sloane *et al.* 1990). This PI has an extensive lateral root system, high root surface area (Goldman *et al.* 1989; Sloane *et al.* 1990; Hudak and Patterson 1996; Pantalone and Rebetzke 1996), and low stomatal conductance (Tanaka *et al.* 2010). In a study that evaluated PI 416937 in high VPD conditions, it reached a maximum transpiration rate near 2.0 kPa (unit of pressure), whereas other genotypes continued to increase transpiration rates at much greater than 2.0 kPa (Fletcher *et al.* 2007). This indicated that water conservation during vegetative growth may be the basis for the slow wilting trait found in PI 416937. PI 471938, a MG V introduction from Nepal, also exhibits the slow wilting trait, but the basis for this trait is unknown (Sadok *et al.* 2012; Bagherzadi *et al.* 2017). PI 471938 was also previously identified as expressing N_2_ fixation tolerance to soil drying (Sinclair *et al.* 2000; Devi and Sinclair 2013; Riar *et al.* 2018). These two plant introductions are being used as sources of slow wilting in applied breeding programs (Devi *et al.* 2014; Carter *et al.* 2016). Two additional MG III PIs were also previously identified that have reduced yield loss under drought stress and delayed leaf wilting (Pathan *et al.* 2014).

The canopy wilting trait has been mapped using linkage and genome-wide association mapping approaches. Charlson *et al.* (2009) mapped four QTL on chromosomes (Chr) 8, 13, 14, and 17 that collectively explained 47% of phenotypic variation in a KS4895 × ‘Jackson’ RIL population (Johnson 1958; Hwang *et al.* 2015a). Eight QTL were identified for canopy wilting under field and greenhouse conditions in Du *et al.* (2009) using 184 RILs derived from Kefeng No. 1 × Nannong1138-2. Using 150 RILs derived from the hybridization of ‘Benning’ (Boerma *et al.* 1997) and PI 416937, seven QTL were identified by Abdel-Haleem *et al.* (2012) that explained 75% of the variation in canopy wilting. Hwang *et al.* (2015b) identified eight QTL clusters that had QTL from at least two of five different RIL populations (93705 KS4895 × Jackson, 08705 KS4895 × Jackson, KS4895 × PI 424140, ‘A5959’ × PI 416937, and Benning × PI 416937) responsible for canopy wilting. With these same populations, Hwang *et al.* (2016) performed a meta-analysis on nine QTL to refine map positions and reduce confidence intervals for the eight QTL clusters reported by Hwang *et al.* (2015b). Kaler *et al.* (2017b) used a genome-wide association analysis of 373 MG IV genotypes to identify 61 single nucleotide polymorphism (SNP) markers associated with canopy wilting, which tagged 51 different genetic loci. Of the 373 genotypes tested, 185 genotypes had lower canopy wilting scores across environments than PI 416937 (Kaler *et al.* 2017b).

There are approximately 170,000 soybean accessions maintained in germplasm collections worldwide, and the USDA maintains a collection of around 20,000 accessions. However, only a limited number of these genotypes have been screened for stress tolerance, and few have been identified as tolerant and used in soybean breeding programs to improve drought tolerance (Carter *et al.* 1999, 2004, 2016; Devi *et al.* 2014; Sinclair *et al.* 2016). Identification of new accessions with beneficial alleles for drought tolerance related traits, including slow canopy wilting, could help in the development of drought tolerant soybean cultivars. To aid in the search for beneficial alleles, the SoySNP50K and SoySNP6K iSelect BeadChips are available for high-throughput genotyping that supports QTL mapping efforts. In addition, the entire USDA soybean germplasm collection has been genotyped with the SoySNP50K iSelect BeadChips (Song *et al.* 2013, 2014, and 2015).

Genome-wide association studies (GWAS) allow for the opportunity to identify the genomic regions for traits of interest by utilizing diverse soybean germplasm and populations. Use of association panels can increase the mapping resolution compared to traditional QTL mapping (Deshmukh *et al.* 2014). Population structure in these panels can occur if some of the genotypes are more related to each other compared to the rest of the population. Failure to correct for population stratification in GWAS models can lead to false positives, especially if the trait of interest is correlated with the structure of the panel (Wang *et al.* 2005). Several studies in soybean have been reported using the GWAS approach with SNP markers for many different traits, such as seed composition (Hwang *et al.* 2014; Vaughn *et al.* 2014; Bandillo *et al.* 2015; Cao *et al.* 2017), salt tolerance (Patil *et al.* 2016; Zeng *et al.* 2017), carbon isotope composition (Dhanapal *et al.* 2015; Kaler *et al.* 2017a), ureide concentration (Ray *et al.* 2015), agronomic traits (Zhang *et al.* 2015; Contreras-Soto *et al.* 2017; Li *et al.* 2017), chlorophyll traits (Dhanapal *et al.* 2016), local adaptation (Bandillo *et al.* 2017), insect resistance (Chang and Hartman 2017), and canopy wilting (Kaler *et al.* 2017b). These studies have provided a useful way to identify potential genomic regions with high resolution and candidate genes or QTL for traits of interest.

The objectives of this study were to: i) evaluate a genetically diverse panel of soybean genotypes in repeated field experiments for canopy wilting, ii) identify new germplasm that possesses the slow canopy wilting trait, and iii) elucidate genomic regions responsible for canopy wilting using an association mapping approach.

## Materials And Methods

### Plant materials and panel selection

When selecting the panel, approximately 600 soybean accessions were chosen initially from the USDA collection based on geographic origin and low annual precipitation. The 600 were truncated to 169 accessions by examining the diversity among the accessions based on SNP genotype profiles. Only PIs with less than 85% similarity to each other based on these SNP genotypes were included in the panel. An additional 40 newly developed breeding lines with enhanced drought-related traits, as well as drought tolerant and susceptible checks, were added to bring the total number of genotypes in the panel to 209. These 209 genotypes were derived from 30 countries and range from MG III-IX. To minimize the maturity effect on the canopy wilting, only canopy wilting scores from 162 MG VI-VIII lines (groups commonly grown in the southeastern USA) were used in analyses in this study.

### Genotype data and quality control

All but nine of the genotypes in the panel were previously genotyped with the SoySNP50K iSelect BeadChips (Song *et al.* 2013). These nine accessions were genotyped using the same procedure at the USDA Soybean Genomics and Improvement Lab in Beltsville, MD (Song *et al.* 2013). Briefly, 15 seed from each of these nine accessions were grown in a single 32 oz. styrofoam cup in a greenhouse at the University of Georgia, Athens, GA, USA. After approximately two weeks, leaf tissue was harvested and bulked in a 50 mL tube. The tissue was then placed in a lyophilizer for two days, and ground into a fine powder using a Geno/Grinder (SPEX SamplePrep, Metuchen, New Jersey, USA). DNA was extracted and then genotyped with the 50K chip. All SNP marker data were downloaded from SoyBase (Grant *et al.* 2010) for the remaining accessions. A total of 42,079 SNP markers were available from these genotyping efforts. The final number employed for the analyses was 34,892 for all but one environment (File S1), with the Kansas 2015 environment employing 34,397 markers after removing markers with minor allele frequencies below 0.05. The number of markers used varied slightly in the Kansas 2015 environment due to the different number of accessions evaluated being slightly lower. The physical positions of Glyma.Wm82.a2 reference genome were used to determine the locations of the SNPs used in the analysis.

### Evaluation of canopy wilting

Canopy wilting was evaluated in Athens, GA, USA in 2015 (GA-15) and 2016 (GA-16) and Salina, KS, USA in 2015 (KS-15) and 2016 (KS-16) in rain-fed field plots after extended periods of little or no rainfall (File S2). Genotypes were planted in two-row plots in each environment in a randomized complete block design with three replications. Experiments were sown on June 16, 2015 (GA-15), June 8, 2016 (GA-16), June 10, 2015 (KS-15), and June 8, 2016 (KS-16). All plots for the four environments were planted with 0.76 m row spacing at a seeding density of 32 seed m^-2^. For GA-15 and GA-16, the plots were 2.43 m in length, and for KS-15 and KS-16 the plots were 3.65 m long.

Wilting was rated in increments of 5 on a scale from 0 to 100: 0 = no wilting present; 20 = slight wilting and some rolling in the top of the canopy; 40 = somewhat severe leaf rolling at the top of the canopy, moderate wilting of leaves throughout the rest of the canopy, and some loss of petiole turgidity; 60 = severe wilting of leaves throughout the entire canopy, with advanced loss of petiole turgidity; 80 = plants with petioles severely wilted and dead leaves throughout much of the canopy; and 100 = plant death. Volumetric water content (VWC) was measured with a single Decagon GS1 soil moisture probe placed approximately 30 cm below the soil surface in one corner of the field plots to measure available soil water at the time the scores were recorded.

Wilting scores in Athens, GA were taken by two raters in 2015 and three raters in 2016. In 2015, a single canopy wilting rating was taken on 29 July (most genotypes in vegetative stages, 17% VWC) for the Athens, GA plots and four ratings were taken in 2016 between 25 August and 16 September (most genotypes in pod filling stage, ∼5–8% VWC). One rater recorded canopy wilting scores for Salina, KS in both years. In 2015, four ratings were taken between 12 August and 28 September (most genotypes in vegetative stage for the first rating and at the pod filling stage for the last rating, ∼18–20% VWC), and in 2016 three ratings were taken between 26 July and 4 August (during flowering, VWC data not available). Mean ratings for an individual plot over dates and raters were employed as the phenotypic wilting score given that correlations between raters and rating dates were generally high (data not shown). All 162 entries were evaluated in GA-15, GA-16, and KS-16. However, because of seed availability and quality, only 142 entries were evaluated in KS-15.

### Population structure analyses

Population structure was determined using fastSTRUCTURE (Raj *et al.* 2014), principal coordinate analysis, and by constructing a dendrogram based on the 50K SNP data. The fastSTRUCTURE program was run in default settings with the simple option testing for subpopulations (K) ranging from K = 2 - 10. As part of the fastSTRUCTURE package, the python script ChooseK was used to choose the number of subpopulations that maximize the marginal likelihood. Principal coordinate analysis was performed using the GAPIT R package (Lipka *et al.* 2012) and visualized with TIBCO Spotfire (TIBCO Software Inc., Palo Alto, CA, USA). The neighbor-joining clustering algorithm in TASSEL version 5.0 (Bradbury *et al.* 2007) was used to build a dendrogram, which was visualized with FigTree (http://tree.bio.ed.ac.uk/software/figtree/).

### Statistical analyses

Analyses of variance (ANOVA) were conducted using PROC GLM in SAS version 9.4 (SAS Institute 2014). A model for canopy wilting scores was created with genotype treated as a fixed effect, and environment, replication within environment, and genotype by environment interaction treated as random effects. Broad-sense heritability was calculated on an entry-mean basis after Holland *et al.* (2010) with the variance components being calculated with PROC MIXED of SAS version 9.4 using a model where all variables were treated as random. Correlations of genotype means were calculated using PROC CORR in SAS version 9.4. Best linear unbiased predictions (BLUPs) were calculated for canopy wilting scores across all environments using JMP Pro (JMP, Version 13, SAS Institute Inc., Cary, NC, USA). The model was built by treating genotype, environment, genotype by environment interaction, and replication within environment as random variables using the Standard Least Squares personality and REML method. For individual environments, only genotype and replication were used and treated as random to calculate BLUPs. Use of BLUP values for each genotype across and within environments helped to account for variation caused by environmental factors and missing data. These BLUPs were used as the phenotype values for subsequent GWAS analyses.

### Genome-wide association analyses

Genome-wide association analyses were performed using Fixed and random model Circulating Probability Unification (FarmCPU) (Liu *et al.* 2016). This R package uses a multiple loci linear mixed model (MLMM) which incorporates the most significant SNP markers as covariates in a modified mixed linear model (MLM), and uses fixed and random effect models iteratively to eliminate confounding between kinship and the markers being tested. This method helps to improve statistical power to detect significant markers associated with a particular phenotype and is computationally efficient. A total of 34,892 genome-wide SNP markers were used for the analysis after removing markers with minor allele frequencies (MAF) below 0.05. The number of markers used varied slightly in the KS-15 environment (34,397 markers) due to a smaller number of accessions evaluated. The differences in accession number affected which SNP markers were included for each genotype file when the MAF of the marker was close to 0.05. Manhattan plots were visualized with the ‘qqman’ R package (Turner 2018) using p-values generated from the FarmCPU output.

A Bonferroni threshold (*P* < 2.83E-07, -log_10_*(P)* > 6.55) is overly strict when the linkage disequilibrium among genetic markers is large, which is generally the case with soybean (Hyten *et al.* 2007). Therefore, a p-value threshold of (*P* < 0.0001; -log_10_*(P)* > 4) was used, which is less stringent than the Bonferroni-corrected threshold, but more stringent than the threshold used in Kaler *et al.* (2017b) and many other soybean GWAS studies using SoySNP50K genotype data. This threshold was used to identify SNPs that were significantly associated with the canopy wilting trait.

Pairwise estimates of Dʹ and r^2^ were calculated by chromosome using Haploview version 4.2 software (Barrett *et al.* 2005). Linkage disequilibrium (LD) blocks were estimated using the Solid Spine of LD option using Dʹ > 0.8 to extend the spine. Significant SNPs associated with canopy wilting were considered part of the same locus (genomic region) controlling the trait if they were in the same LD block. Allelic effects were calculated by taking the difference in mean canopy wilting score between the two alleles at a particular SNP, and the direction, negative or positive, of the allelic effect estimates were relative to the alphabetical order of the nucleotides at each particular marker. For example, if the nucleotides at a particular SNP are “A” and “C”, then a positive (above zero) allelic effect (*e.g.*, 3) indicates that possessing the “C” allele will increase the canopy wilting score by three, which is unfavorable. A negative (below zero) effect value indicates that a line possessing the second nucleotide alphabetically for this SNP would have a lower canopy wilting score, which is favorable. For this study, the allelic effects are based on BLUP values, not actual canopy wilting scores, so while the overall effect is relevant, it does not directly apply to raw canopy wilting scores. The amount of phenotypic variation explained (R^2^) for all significant SNPs in a given environment was calculated using a simple linear regression in R with lm(BLUP ∼ SNP_1_ + SNP_2_ + …).

Breeding values for accessions evaluated were calculated by adding the allelic effects for all SNPs significantly associated (*P* < 0.0001; -log_10_*(P)* > 4) with canopy wilting in each individual environment and with the across all environments BLUPs. Breeding values from across individual environments (GA-15, GA-16, KS-15, and KS-16) were also summed. The allelic effect for a given accession was considered below zero (favorable) if the allele contributed to lower canopy wilting scores. In contrast, if the allele increased canopy wilting score, it was considered an above zero (unfavorable) value. Therefore, a more negative breeding value indicated an accession had a sum of allelic effects across all significant SNPs that was more favorable toward reduced canopy wilting. If the allele at a particular SNP was heterozygous or missing for a genotype, it was omitted in the breeding value calculation.

### Candidate gene identification

SNPs from the GWAS that met the threshold of -log_10_*(P)* > 4 were used to identify nearby candidate genes. Candidate genes and their functional annotation were identified using the Glyma2.1 gene models in SoyBase for models within plus or minus 10 kb of the SNP physical position.

### Data availability

SNP marker genotypes for accessions included in the association panel are in File S1 and canopy wilting scores are in File S2. All other datasets generated and/or analyzed for this study are not publicly available, but are available from the corresponding author based on a reasonable request. Supplemental material available at figshare: https://doi.org/10.25387/g3.11441340.

## Results

### Canopy wilting

**Figure 1:**
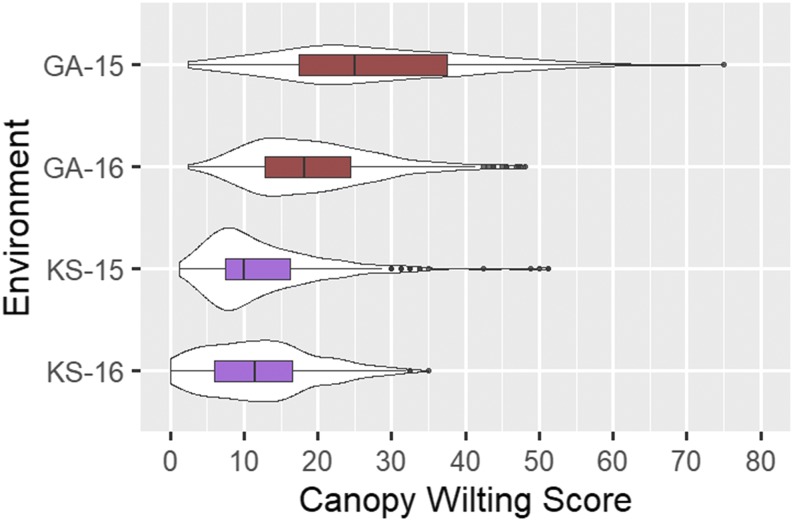
Violin plots with boxplots inside showing the distribution of canopy wilting scores. Environments are named as Location-Year, with Georgia (GA) and Kansas (KS) as locations, and 2015 (15) and 2016 (16) as years.

Substantial variation for canopy wilting was observed among the genotypes within the panel across the four environments tested. In general, canopy wilting scores were higher (more severe wilting) in Athens, GA compared to Salina, KS in both 2015 and 2016 (Figure 1). Genotypes, environments, and their interactions were statistically significant (*P* < 0.05) for canopy wilting scores (Table 1). Correlations (data not shown) of canopy wilting scores based on genotype means among the environments ranged from r = 0.42 (KS-15/KS-16) to r = 0.65 (GA-15/KS-16). Broad sense heritability of canopy wilting on an entry-mean basis for each environment was 60% (GA-15), 73% (GA-16), 30% (KS-15), 72% (KS-16), and 34% across all environments.

**Table 1:** Summary of analyses of variance (ANOVA) for effects of genotype (G), environment (E), and their interaction based on canopy wilting scores. The G × E MS was used as the denominator of the F Value for significance testing

Source	DF	F Value	*P* > F
Genotype (G)	161	10.1	<0.0001
Environment (E)	3	648.2	<0.0001
G × E	483	2.1	<0.0001

The 162 genotypes were ranked from lowest to highest canopy wilting score within each environment, and then over the four environments (Tables 2 and S1). Numerically, 78 genotypes exhibited less wilting than the slow canopy wilting check, PI 416937. Thirty-eight and 44 lines exhibited numerically greater wilting than fast wilting cultivars Benning and Hutcheson, respectively (Table S1).

**Table 2:** Canopy wilting scores for the 10 genotypes with the lowest and highest scores based on mean ranking across environments along with two check genotypes. Each environment was ranked individually, and the mean of those rankings was used to rank all of the 162 genotypes tested. Canopy wilting scores shown are the mean of all replications within each respective environment. A full table of all accessions tested and their canopy wilting scores is provided in the supplementary materials (Table S1)

				Canopy Wilting Score							
Accession	Name	Country*^a^*	MG*^b^*	ALL-PANEL	GA-15-PANEL	GA-16-PANEL	KS-15-PANEL	KS-16-PANEL	Rank	Beneficial Alleles*^c^*	Breeding Value*^d^*
Slow wilting											
PI603535	Hei zong huang dou	China	VIII	6	10	6	6	1	1	18	−31.80
PI603513A	Xiao niu mao huang	China	VIII	7	9	7	6	5	2	23	−19.12
PI603529	Hei huang dou	China	VIII	8	12	10	—	2	3	15	15.55
PI603513B	—	China	VIII	8	13	10	—	3	4	28	−17.33
PI603534A	Da niu mao huang	China	VII	8	7	12	8	5	5	26	−15.62
PI219698	Kulat	Pakistan	VI	10	18	11	—	3	6	25	−32.46
PI532458	Ba yue bao	China	VIII	10	23	10	3	3	7	22	−25.01
PI269518B	(Koolat)	Pakistan	VI	11	21	9	—	3	8	22	−25.16
PI567405	Wei zi dou	China	VI	9	10	12	7	6	9	27	5.93
PI603521	Huang dou	China	VIII	10	18	8	9	5	10	16	−14.86
Checks											
PI416937	Houjaku Kuwazu	Japan	VI	18	39	16	8	11	79	23	−34.10
PI595645	Benning	United States	VII	23	38	24	11	20	132	23	−1.84
Fast wilting											
PI424131	Buffalo	Zimbabwe	VII	27	36	26	23	25	153	24	−28.20
PI430737	Oribi	Zimbabwe	VII	31	49	27	—	16	154	21	−11.27
PI567377B	(Ba yue zha)	China	VI	34	63	32	13	26	155	22	−17.65
PI159096	41S77	South Africa	VII	31	52	29	25	17	156	20	−33.96
PI381663	Kakira 1	Uganda	VI	35	55	45	20	20	157	25	−34.39
NCC06-1090	—	United States	VI	32	39	39	24	28	158	11	13.74
PI639573	—	Burundi	VIII	33	39	37	30	25	159	26	−34.27
PI599333	Musen	United States	VI	33	53	32	20	27	160	16	7.09
PI417562	54.S.30 DL/64/185	South Africa	VI	36	46	36	36	25	161	17	−11.48
PI330635	—	South Africa	VII	39	53	40	—	25	162	23	−20.70

a Country of origin of the accession based on GRIN data.

b Maturity group.

c Number of alleles from all significant SNPs with an effect that reduces canopy wilting score.

d Breeding value determined by adding the allelic effects for all significant SNPs individually by environment, and then summing the breeding values across individual environments.

### Population structure

**Figure 2:**
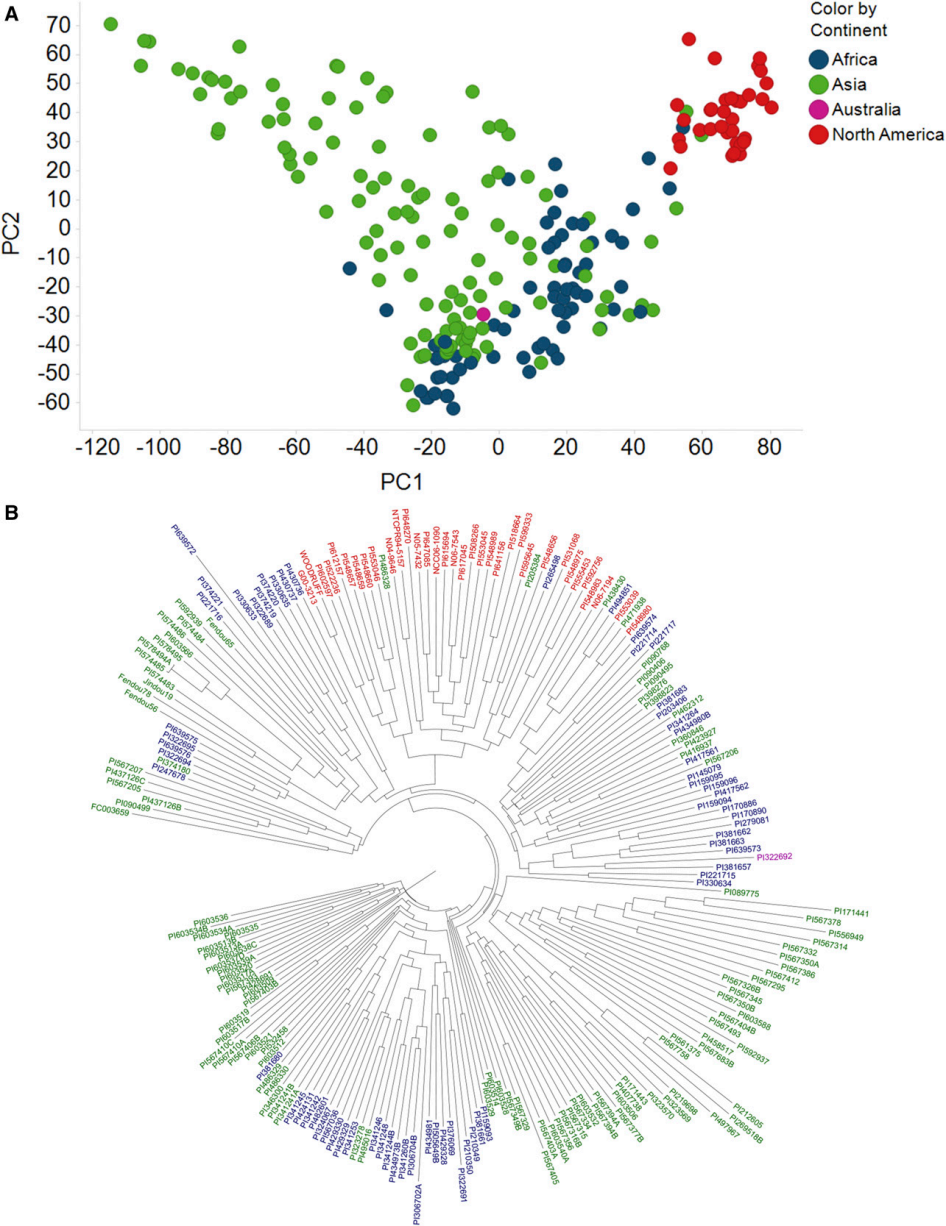
(A) Plot of first and second principal coordinates for a diverse panel of soybean accessions evaluated in drought tolerance related studies. Each individual soybean genotype is colored by their continent of origin. (B) Dendrogram using neighbor joining clustering algorithm in TASSEL visualized in FigTree. Genotypes are colored by their continent of origin: red = North America, blue = Africa, green = Asia, and purple = Australia.

All population structure analyses were conducted using the full panel of 209 genotypes initially selected from MG III to IX for other drought tolerance related studies conducted in Steketee *et al.* (2019), which include the 162 genotypes evaluated for canopy wilting in this study. The first two principal coordinates were visualized and colored by continent of origin (Figure 2A). All of the North American lines represent genotypes from the USA, and the majority (88/118) of Asian genotypes were from China. The genotypes of U.S. origin were more tightly clustered than were genotypes from China (Figure 2A). The first four principal coordinates explained approximately 19% of the variation in the data set, and were used as covariates in the GWAS model to help correct for potential population stratification (Figure S1). The North American genotypes had shorter genetic distances between accessions compared to Asian and African genotypes based on the neighbor joining dendrogram analysis, indicating they are more closely related, which concurs with the principal coordinates analysis. Genotypes from Asia tended to group close to one another based on this analysis, but also intermixed with lines of African origin (Figure 2B). A continuous increase in marginal likelihood with increasing K was observed for the fastSTRUCTURE analysis, meaning as each sequential K value (increasing from 2 to 10) was tested it was deemed the K value that best characterized the population structure of the panel. This indicated that little structure was apparent for this population, because fastSTRUCTURE was not able to settle on an optimal K value to describe this panel within K = 2 - 10 (Westbrook *et al.* 2015) (Figure S2).

### GWAS of canopy wilting trait

**Figure 3:**
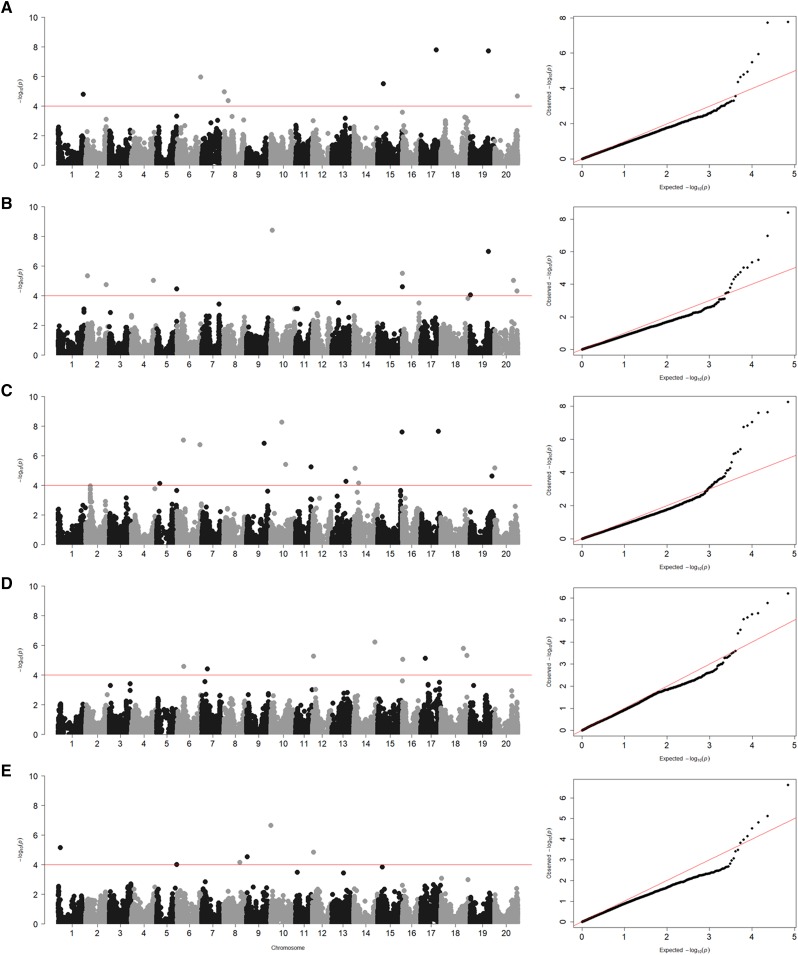
Genome-wide Manhattan plots for (A) ALL, (B) GA-15, (C) GA-16, (D) KS-15, and (E) KS-16. The X-axis is the genomic position of SNPs by chromosome across the soybean genome, and the Y-axis is the -log_10_ of the p-values obtained from the GWAS model. Significance threshold -log_10_*(P)* > 4 (red line). The quantile-quantile (QQ) plots to the right of each Manhattan plot show the expected *vs.* observed p-values of each SNP tested in the GWAS models.

Across and within environments, 45 unique SNPs were identified that tagged 44 loci associated with canopy wilting from the GWAS analysis (Figure 3 and Table 3). One of these SNPs (ss715634688) on Chr 19 was significant (*P* < 0.0001; -log_10_*(P)* > 4) both in individual environments and with the BLUP value calculated using the canopy wilting scores across all environments. One additional region, locus 32 on Chr 16 was identified in two of the four environments.

**Table 3:** SNPs that met significance level of -log_10_*(P)* > 4 for the GWAS of canopy wilting

Locus*^a^*	Chr.*^b^*	Pos.*^c^*	SNP	-log_10_*(P)*	MAF*^d^*	Effect*^e^*	Env*^f^*	SoyBase QTL*^g^*
1	1	4015639	ss715579324	5.13	0.27	−1.62	KS-16	
2	1	51961463	ss715580187	4.78	0.09	2.13	ALL	
3	2	3165348	ss715581823	5.34	0.06	−3.65	GA-15	Canopy wilt 3-4, 3-8, 3-11, 6-1; mqCanopy wilt-001
4	2	42073473	ss715582842	4.73	0.37	1.97	GA-15	Canopy wilt 6-3
5	4	46096228	ss715588277	5.02	0.38	−1.97	GA-15	
6	5	6207961	ss715592288	4.13	0.06	−3.19	GA-16	Canopy wilt 3-5
7	5	41387548	ss715591700	4.45	0.21	1.91	GA-15	
8	6	13090474	ss715592991	7.05	0.06	2.97	GA-16	
9	6	14258126	ss715593189	4.56	0.06	1.51	KS-15	
10	6	47633030	ss715594738	6.75	0.28	−1.87	GA-16	Canopy wilt 3-12
11	6	49189084	ss715595012	5.94	0.37	1.50	ALL	
12	7	11177483	ss715596171	4.40	0.32	0.71	KS-15	
13	8	1699023	ss715599875	4.94	0.24	1.40	ALL	
14	8	9837263	ss715602901	4.36	0.15	−1.42	ALL	
15	8	34471238	ss715601484	4.14	0.20	1.79	KS-16	
16	9	1769730	ss715603168	4.53	0.18	−1.93	KS-16	
17	9	36942176	ss715603680	6.84	0.31	−2.01	GA-16	
18	10	270252	ss715606054	6.63	0.47	1.81	KS-16	
19	10	3598580	ss715606348	8.41	0.41	3.20	GA-15	
20	10	23376136	ss715605804	8.26	0.27	−3.31	GA-16	
21	10	30831897	ss715606157	5.41	0.15	3.43	GA-16	
22	11	31929823	ss715610250	5.25	0.48	−1.45	GA-16	
23	12	2053039	ss715611755	5.26	0.08	−1.38	KS-15	
24	12	2839426	ss715612002	4.82	0.35	−1.43	KS-16	
25	13	29459954	ss715614803	4.25	0.10	−1.89	GA-16	
26	14	3078346	ss715618273	5.13	0.43	1.55	GA-16	Canopy wilt 1-2
27	14	10057919	ss715617366	4.15	0.07	2.76	GA-16	
28	14	43597753	ss715618915	6.21	0.36	−0.90	KS-15	
29	15	12437556	ss715620442	5.49	0.34	1.29	ALL	
30	15	50499617	ss715622647	7.59	0.24	2.16	GA-16	
31	15	51622014	ss715622805	4.60	0.23	−1.75	GA-15	
32	16	164715	ss715623538	5.49	0.48	1.81	GA-15	
	16	517535	ss715625192	5.04	0.09	−1.29	KS-15	
33	17	9384325	ss715628378	5.12	0.43	0.75	KS-15	Canopy wilt 1-3, 3-10, 3-13; mqCanopy wilt-006
34	17	31794022	ss715626726	7.78	0.12	2.51	ALL	Canopy wilt 5-2
35	17	36227875	ss715626968	7.64	0.09	3.47	GA-16	
36	18	46860521	ss715631153	5.78	0.13	−1.26	KS-15	
37	18	54371258	ss715632037	5.31	0.09	1.47	KS-15	
38	19	809326	ss715636293	4.04	0.28	−1.80	GA-15	
39	19	38109922	ss715634688	7.73	0.15	−2.16	ALL	Canopy wilt 2-7, 4-3
	19	38109922	ss715634688	6.98	0.15	−3.05	GA-15	Canopy wilt 2-7, 4-3
40	19	45307395	ss715635460	4.61	0.34	1.47	GA-16	Canopy wilt 5-4, 6-2
41	20	294010	ss715637218	5.17	0.46	1.55	GA-16	
42	20	39013106	ss715637991	5.03	0.36	−1.56	GA-15	
43	20	46438247	ss715638748	4.30	0.46	−1.71	GA-15	
44	20	47435005	ss715638900	4.65	0.48	−1.46	ALL	

a If multiple SNPs were identified in the same linkage disequilibrium (LD) block they were deemed part of the same locus (genomic region).

b Chromosome.

c Glyma.Wm82.a2 physical position.

d Minor allele frequency.

e Allelic effects were calculated by taking the difference in mean canopy wilting score between the two alleles at a particular SNP, and the direction, negative or positive, of the allelic effect estimates are relative to the alphabetical order of the nucleotides at each particular marker.

f Environment written as location-year-population.

g Canopy wilting QTL identified on SoyBase in which loci from our study are located within.

The quantile-quantile (QQ) plots follow the expected diagonal, with a sudden uptick for statistically significant (*P* < 0.0001; -log_10_*(P)* > 4) SNP markers in each environment (Figure 3). This linear pattern of expected *vs.* observed p-values also does not have a slope greater than one, which indicates the first four principal coordinates included in the GWAS model adequately accounted for population stratification in this panel of genotypes. The deviation of the markers from this diagonal occurs at or greater than –log_10_*(P)* = 4 in each environment, which was the threshold we used to determine if a SNP marker was significantly associated with the canopy wilting trait.

Allelic effects across all significant (*P* < 0.0001; -log_10_*(P)* > 4) SNPs ranged from -3.65 to 3.47 (Table 3). The R^2^ for all significant SNPs in a given environment was 54% (GA-15), 45% (GA-16), 53% (KS-15), 30% (KS-16), and 36% (ALL). The number of beneficial alleles each genotype possessed was determined by counting the number of alleles with effects that reduced canopy wilting score from all the significant SNPs. Overall, the number of beneficial alleles ranged from 11 to 31. The 10 slowest wilting genotypes had 15 to 27 beneficial alleles, while the 10 fastest wilting genotypes had 11 to 26 beneficial alleles (Table 2 and Table S1). Summed breeding values across the individual environments ranged from -42.32 to 17.17 overall (Table 2 and Table S1). Negative (below zero) breeding values indicate that the genotype had a sum of allelic effects across the significant SNPs that was more favorable toward reduced canopy wilting scores. Positive (above zero) breeding values indicate that the genotype had a sum of allelic effects across the significant SNPs that was less favorable toward reduced canopy wilting scores.

### Identification of candidate genes for the canopy wilting trait

The median distance between SNP markers used in the GWAS was 9 kb, and the mean distance was 26 kb. Although identifying all gene models in LD with significant SNPs would be ideal, we focused our efforts on models in close proximity (within plus or minus 10 kb), which approximately spans this distance between markers. Eighty-seven candidate genes were found within plus or minus 10 kb of the 45 significant (*P* < 0.0001; -log_10_*(P)* > 4) SNPs for the GWAS (Table S2).

## Discussion

### Canopy wilting

In this study, we evaluated 162 soybean genotypes in four environments for canopy wilting score after extended periods of drought stress, and most of these genotypes were never evaluated previously for drought tolerance related traits. There was substantial genetic variation for canopy wilting within each environment (Figure 1). Canopy wilting is a complex, quantitative trait (Charlson *et al.* 2009; Hwang *et al.* 2016), and our phenotypic data further confirm this notion.

Slow wilting PI 603535, a MG VIII accession from China, had a mean score of 6 across all environments. Eight of the 10 genotypes with the slowest wilting ranking originated from China. Among the fast wilting genotypes in the panel, PI 330635, a MG VII accession from South Africa, had a mean canopy wilting score of 39 across environments (Table 2). PI 416937 was included as a slow wilting check in the studies, and many accessions had lower mean wilting scores (less wilting) than this check genotype. Seventy-eight genotypes had lower canopy wilting scores than PI 416937 (Table S1). However, the mean canopy wilting score across all environments evaluated for PI 416937 was 18, which is also a relatively low score (Table S1). These newly identified PIs could be germplasm sources as parents that could be exploited by soybean breeders to improve the canopy wilting trait, especially the accessions with favorable alleles different than PI 416937, and with negative breeding values. In Kaler *et al.* (2017b), 185 of the 373 genotypes they tested had scores lower than PI 416937. This study and Kaler *et al.* (2017b) demonstrate there is more variation and potential for improvement of the canopy wilting trait than previously reported, but testing these new slow wilting genotypes in more environments is necessary to further confirm they will consistently exhibit this trait in different locations and drought stress severities.

### Physiological mechanisms for canopy wilting and relationship to other traits

Slow canopy wilting could lead to less yield reduction during drought stress in soybeans. A previous study proposed three different combinations of physiological mechanisms that could lead to delayed canopy wilting (Ries *et al.* 2012). One is a combination of high water use efficiency (WUE), high radiation use efficiency (RUE), and conservation of soil moisture. Genotypes in this group would utilize transpired water for biomass production more efficiently, and higher RUE would be expected in both drought stressed and optimal growing conditions. The second combination is low stomatal conductance, low RUE, low WUE, and conservation of soil moisture. The genotypes in this group would have low transpiration which would reduce potential photosynthetic capacity, and would be better at conserving water during drought stress conditions. However, this second combination of physiological attributes could reduce overall yield potential, especially in well-watered environments. Deeper rooting is a third mechanism that could delay canopy wilting in soybean (Ries *et al.* 2012). Given these advantages and trade-offs for different physiological traits, identifying soybean germplasm with the optimal combination to reduce canopy wilting during drought stress will be different depending on the target environment.

Much like canopy wilting in soybean, evaluation of other crops for drought tolerance commonly uses secondary traits for indirect selection which can show relationships with yield under stressed conditions. Leaf rolling reduces exposed leaf area, and thereby decreases transpiration and reduces light interception, and can be observed in crops such as maize, rice, and wheat. The earlier leaf rolling occurs in a given day or longer duration of rolling indicates the plant is experiencing more stress (Rauf *et al.* 2016). Therefore, ratings and selections can be made to identify plants with reduced leaf rolling during drought periods to improve drought tolerance. In maize, another trait that is evaluated to improve drought adaptation is the anthesis-silking interval (ASI). This trait is negatively correlated with grain yield under drought conditions, and has been a breeding target due to the ease of measurement and moderate heritability (Tuberosa 2012). Additional traits such as stay green, root architecture, and canopy temperature depression can impact a plant’s ability to tolerate drought stress and have been evaluated in a number of crop species (Tuberosa 2012). Slow canopy wilting in soybean is potentially related to other secondary physiological mechanisms that can be evaluated to improve our understanding of soybean drought physiology and potentially improve yield under stressed conditions.

### Relationship of canopy wilting, days to flowering, and maturity group

Canopy wilting scores were overall higher in Georgia compared to Kansas in both 2015 and 2016 (Figure 1). Given that the genotypes we evaluated consisted of genetically diverse genotypes (most of which were plant introductions) with some variation in phenology (flowering time, height, root mass, etc.) these scores could potentially be affected by varying degrees of competition for water resources from neighboring plots due to these factors.

Days to flowering (DTF) was recorded in the GA-15 and GA-16 environments as the number of days from planting until 50% of the plants in a plot reached the R1 (first bloom) stage of development. Maturity groups (MG) for all accessions were obtained from the USDA GRIN website or were provided by the breeder who developed the line. Correlations between wilting score and MG (r = -0.34 to -0.05), and wilting score and DTF (r = -0.30 – 0.00) in single environments were relatively low. Across all environments, the correlation for wilting score and MG was r = -0.20 and for wilting score and DTF was r = -0.22. There did not appear to be a relationship between mean rank across environments and MG or DTF in this study (Figure S3). In our study, canopy wilting was primarily evaluated during the early reproductive growth stages, with the exception of GA-15 and first rating in KS-15, which were rated during the late vegetative growth stages for most genotypes. Water stress during the early reproductive growth stages has the greatest impact on reducing soybean yield from plants producing fewer pods, and in turn, less seed (Manavalan *et al.* 2009). Therefore, slow canopy wilting during reproductive growth stages could be a good indicator of drought tolerance and ability to maintain yield potential during water stress.

### Genotype by environment interaction and heritability

Although genotype by environment interactions were significant (*P* < 0.05) (Table 1) and the severity of wilting experienced in the four environments varied (Figure 1), the correlations between wilting scores across environments were relatively high (r = 0.42-0.65), indicating that the genotypes tested from this panel wilted similarly across environments. Heritability across environments was also moderate to high, with heritability comparable to those observed in previously canopy wilting QTL mapping and GWAS studies (Charlson *et al.* 2009; Abdel-Haleem *et al.* 2012; Hwang *et al.* 2015b; Kaler *et al.* 2017b).

### Population structure analyses with panel

Soybean was first domesticated in China (Hyten *et al.* 2006), and the accessions of Chinese origin from the panel had the least tight cluster in the principal coordinates plot compared to other countries, and exhibited the greatest distance between accessions in the dendrogram, indicating they had the most diversity of the accessions tested (Figure 2). Lines from the USA were tightly clustered in the principal coordinates plot, and had short distances apart from one another in the dendrogram (Figures 2). The genetic base used in North American soybean breeding has been characterized as being narrow, with only a few common ancestors explaining the majority of diversity for these breeding materials (Carter *et al.* 2004). The tight clustering based on principal coordinates and short distance between accessions in the dendrogram is a reflection of this narrow genetic diversity of elite U.S. soybean breeding lines. Given that the panel of soybean genotypes used for this study was explicitly chosen to be genetically diverse based on genome-wide 50K SNP data, the lack of ability for fastSTRUCTURE to select an optimal number of K groups was expected. Based on the combination of the results of these population structure analyses, we determined that this panel had little or moderate population structure present that would affect the GWAS and cause false positives. As is commonly done with association mapping, we did include the first four principal coordinates in our GWAS model as a way to help reduce the possibility of potential population structure affecting the mapping results (Price *et al.* 2006).

### Comparisons of genetic mapping for canopy wilting to previous studies

We identified 45 unique SNPs that tagged 44 loci that are associated with canopy wilting using a genome-wide association mapping approach. One of these SNPs was found both in an individual environment and when using the BLUP value calculated using the canopy wilting scores across all environments. On Chr 16, two physically close SNPs found in two different environments within the same LD block were significantly associated with canopy wilting. Overall, significant SNPs were identified on 19 of the 20 soybean chromosomes, with only Chr 3 not having any marker-trait associations (Table 3). The R^2^ for all significant SNPs in a given environment was 54% (GA-15), 45% (GA-16), 53% (KS-15), 30% (KS-16), and 36% across all environments.

Several reports of QTL or genomic regions that control canopy wilting in soybean have been previously reported, and many are numbered with their approximate physical locations on the SoyBase website. For the GWAS results, Loci 3 is within the QTL interval for a meta-QTL identified in Hwang *et al.* (2016). Loci 4, 6, 10, 34, and 40 were within QTL found in Hwang *et al.* (2015b). Loci 26 and 33 are within Canopy wilt 1-2 and Canopy wilt 1-3 QTL identified, respectively, in Charlson *et al.* (2009). On Chr 19, Loci 39 was within the Canopy wilt 2-7 QTL identified in Abdel-Haleem *et al.* (2012). Of the 45 SNPs identified from GWAS associated with canopy wilting in the this study, a total of nine were found in or near the same location as canopy wilting QTL identified from the linkage mapping studies described above (Table 3). The overlapping regions and consistent QTL across mapping studies help provide validation that these loci are associated with the canopy wilting trait and could be the targets of improvement efforts.

**Figure 4:**
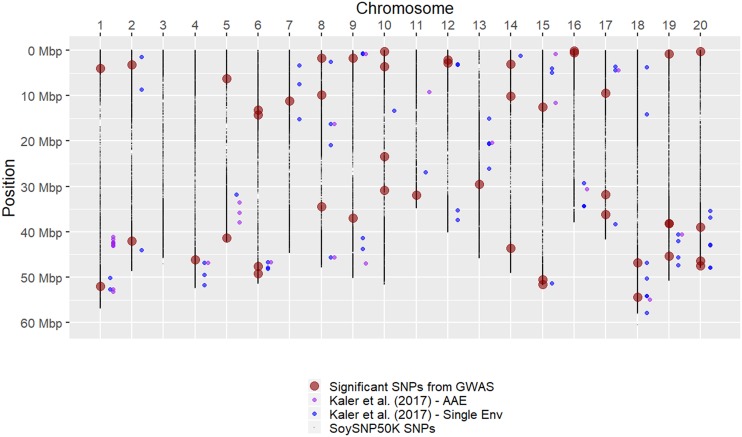
Location and comparison of SNPs significantly associated with canopy wilting based on association mapping results. Physical positions are based on the Glyma.Wm82.a2 version of the soybean genome. SNPs identified in GWAS that met -log_10_*(P)* > 4 significance threshold are shown as large red colored circles. Average of all environments (AAE) and single environment (Env) significant SNPs from Kaler *et al.* (2017b) are shown as purple and blue circles, respectively. Position locations were converted from version 1 to 2 of the soybean genome assembly for the Kaler *et al.* (2017b) SNPs, so that comparisons were made using the same physical positions. ss715637687 found in AAE for Kaler *et al.* (2017b) is not in version 2 of soybean genome assembly, and therefore not included in this comparison.

Kaler *et al.* (2017b) used an association mapping approach to identify 61 SNP markers tagging 51 different loci for canopy wilting. The significant SNPs found in our study and Kaler *et al.* (2017b) were compared (Figure 4). Twelve SNPs tagging 12 genomic regions were found on Chr 1, 4, 6, 9, 12, 15, 18, 19, and 20 in our study that are near SNPs identified in Kaler *et al.* (2017b). The main difference in our study compared to Kaler *et al.* (2017b) is that later maturity group soybeans (VI-VIII *vs.* IV) were used in our study. The genomic regions that are consistent across maturity groups and many different environments show promise as selection targets for improving canopy wilting under drought stress. Directing research efforts toward the genomic regions found in common between the current and previous GWAS studies could yield favorable alleles for the improvement of the canopy wilting trait in soybean. Because many genomic regions were identified, a simple marker-assisted selection approach for improvement of this trait may not be feasible. An alternative, and perhaps more effective, approach would be to utilize genomic selection with whole genome markers to improve canopy wilting in applied breeding programs.

### Candidate genes at canopy wilting significant genomic regions

The SNP with the greatest absolute allelic effect (3.65) was found on Chr 2 (ss715581823) and has a MAF of 0.06 (Table 3). This SNP is located near Glyma.02g034000, which has an aldehyde dehydrogenase annotation (Table S2). Aldehyde dehydrogenase genes have been previously shown to play a role in response to abiotic stresses of soybeans, and can be highly induced by drought stress in soybean leaves (Wang *et al.* 2017). Loci 12 (Glyma.07g110100) and 33 (Glyma.17g118400) have gene models which encodes a RING superfamily protein (Table S2), and locus 33 was co-located with previously identified canopy wilting QTL. RING-type E3 ubiquitin ligases including DREB2A-interacting proteins DRIP1 and DRIP2 have been previously shown to play a role in drought response (Qin *et al.* 2008). GmRFP1 functions as a RING-type E3 ubiquitin ligase and is down-regulated by drought and cold stress, but is induced by ABA and salt stress suggesting it may be involved in abiotic stress response (Du *et al.* 2010). Given their relationship with drought stress response and improvement, these gene models could be targeted for understanding and improving canopy wilting in soybean.

## Conclusions

Using 162 genetically diverse maturity group VI-VIII soybean genotypes, a genome-wide association mapping approach identified 45 unique SNPs tagging 44 loci that are significantly associated with the canopy wilting trait. One of these SNPs was identified in an individual environment, as well as across environments. Of these 45 SNPs, nine were found in or near the locations as previous canopy wilting QTL identified from other linkage mapping studies. In addition, 12 SNPs mapped to 12 genomic regions on 11 chromosomes were found near regions identified in a previous association mapping study for canopy wilting. Candidate genes located at these genomic regions were identified that could help to understand the functions of these genes to improve canopy wilting in soybean. The genomic regions discovered across environments, in addition to the new slow wilting germplasm identified with favorable alleles, can be exploited by breeders to improve soybean drought tolerance.
